# Cerebral derailment after myocardial infarct: mechanisms and effects of the signaling from the ischemic heart to brain

**DOI:** 10.1007/s00109-021-02154-3

**Published:** 2021-10-21

**Authors:** Paolo Gelosa, Laura Castiglioni, Joanna Rzemieniec, Majeda Muluhie, Marina Camera, Luigi Sironi

**Affiliations:** 1 Department of Pharmaceutical Sciences, University of Milan, 20133 Milan, Italy; 2 Centro Cardiologico Monzino, 20138 Milan, Italy

**Keywords:** Myocardial infarct, Stroke, Neuroinflammation, EVs, miRNAs

## Abstract

Myocardial infarction (MI) is the leading cause of death among ischemic heart diseases and is associated with several long-term cardiovascular complications, such as angina, re-infarction, arrhythmias, and heart failure. However, MI is frequently accompanied by non-cardiovascular multiple comorbidities, including brain disorders such as stroke, anxiety, depression, and cognitive impairment. Accumulating experimental and clinical evidence suggests a causal relationship between MI and stroke, but the precise underlying mechanisms have not yet been elucidated. Indeed, the risk of stroke remains a current challenge in patients with MI, in spite of the improvement of medical treatment among this patient population has reduced the risk of stroke. In this review, the effects of the signaling from the ischemic heart to the brain, such as neuroinflammation, neuronal apoptosis, and neurogenesis, and the possible actors mediating these effects, such as systemic inflammation, immunoresponse, extracellular vesicles, and microRNAs, are discussed.

## Introduction

Recent research in cardiological and neurological fields has shown that pathophysiological processes, once considered to lead solely cardiovascular or neurological manifestations respectively, may instead concomitantly affect both systems. Indeed, substantial available evidence has indicated a multitude bidirectional connection between the cardiovascular and central nervous systems [1–3]. In 1985, the term “neurocardiology” was coined for the first time to describe this new interdisciplinary area, which examines the interaction between the cardiovascular and autonomic nervous systems in pathological states [4].

In this context, accumulating clinical and experimental studies suggest a causal relationship between myocardial infarction (MI) and brain pathological alterations. MI and subsequent revascularization therapies could lead to transient reduction in cerebral blood flow, thereby damaging the brain [5]. It has also been argued that vascular inflammation, which is common features of MI patients, could be involved in the induction of depression symptoms [6]. Patients with MI have a high prevalence of behavioral disorders, such as anxiety and depression [7–9] and several associated symptoms, including cognitive deficits [10, 11]. In particular, a recent population-based cohort study showed that patients with MI exhibited a significantly higher risk of anxiety-like disorders (adjusted hazard ratio = 5.06) and depressive disorders (adjusted hazard ratio = 7.23) than those without MI, during the first 2 years of follow-up [9]. A large meta-analysis comparing patients with or without depression after MI showed that depression was associated with a 2.7-fold increased risk of cardiac-related death, a 2.3-fold increased risk of all-cause death, and a 1.6-fold increased risk of cardiovascular events in the 2 years after an acute MI [12]. Similarly, a recent meta-analysis, including 16 studies that enrolled patients with established acute MI, showed that MI patients with anxiety (prevalence ranged from 5.5 to 58.2%) had a significant long-term poorer prognosis (risk ratios = 1.27) and increased long-term major adverse cardiac events (MACEs) (risk ratios = 1.54) than those without anxiety [13]. On the other side, the casual relationship between MI and dementia is instead not fully demonstrated. In fact, only two studies have examined the risk of dementia after MI, but with equivocal findings [14, 15]. A case–control study failed to demonstrate a clear association between dementia and MI [14], whereas a cohort study showed an increased risk for patients with unrecognized MI, but not for patients with recognized MI [15].

Several studies have also demonstrated the association between MI and stroke [16]. These two diseases could evolve in parallel since they share the same risk factors, such as hypertension, diabetes, arrhythmias (including atrial fibrillation), high cholesterol levels, smoking, and chronic kidney disease [17]. At the same time, MI is considered one of the etiological causes of stroke [18]. It has been observed that patients with MI have a higher risk of stroke in the first 4 weeks immediately after acute MI than in the corresponding period of 4 weeks to 1 year after MI, and this risk remains high for the first 12 weeks [19, 20]. Of note, the risk of ischemic stroke was similarly elevated for up to 12 weeks for both ST-elevation myocardial infarction (STEMI) and non-STEMI [20], while the cumulative 3-month and 1-year ischemic stroke incidence were higher among patients with coronary artery bypass surgery (CABG) than among patients without CABG [21]. Over the last decade, the relative risk of ischemic stroke within 30 days [22] and 1 year [23] after acute MI has decreased by about 10% and 20% respectively, likely due to increased use of reperfusion therapies and more intense treatment with statins, acetylsalicylic acid, and P2Y12 inhibitors [21–23]. A recent study demonstrated that unrecognized MI, which makes up between one-third to one-half of all MI events [24], is also associated with an increased risk of stroke [25]. Relevantly, stroke following MI impairs the overall prognosis, since patients with ischemic stroke after acute MI have higher morbidity and mortality rates both in the short and long terms than patients without stroke [26]. In this respect, a recent large retrospective cohort study showed that the perspective of 1‐year mortality was about 15% higher for patients with acute MI plus stroke (51.5%) than for those with MI without stroke (37.1%) [20].

Despite this evidence, the precise underlying relationship between MI and stroke has not yet been elucidated. It is likely that the mechanisms could be different for early and late ischemic stroke after MI. Early ischemic stroke may be originated from embolization of blood clots in the left atrium after atrial fibrillation, or from mural thrombus formed in hypokinetic segments of the left ventricle [16], while late ischemic stroke may be caused by the presence of the mutual underlying risk factors. Instead, hemorrhagic stroke may be induced by antithrombotic medication to prevent the reoccurrence of MI. In addition, the incidence of both ischemic and hemorrhagic strokes was higher with percutaneous mechanical circulatory support device use in comparison to those without device in patients with STEMI and cardiogenic shock, suggesting that patients with devices may be hemodynamically sicker and require increased use of anticoagulation [27].

Overall, these findings suggest that the etiopathology of stroke after MI is a complex process. MI may result from a generalized and severe atherosclerotic disease associated with a systemic inflammation and alterations in the function of the neurocardiac axis [28] that, in turn, may increase the ischemic stroke risk. Although the improvement of medical treatment for hypercholesterolemia and pro-thrombotic status among patients with MI has reduced the risk of stroke, this one remains a current challenge. Growing evidence, especially from experimental studies and still to be consolidated, suggest that additional mechanisms could be involved in stroke development after MI. Thus, in this review, we will focus on these other possible signaling mechanisms relayed from the ischemic heart to the brain and the resulting alterations in brain functioning.

## Mechanisms of heart–brain interaction after MI

**Fig. 1:**
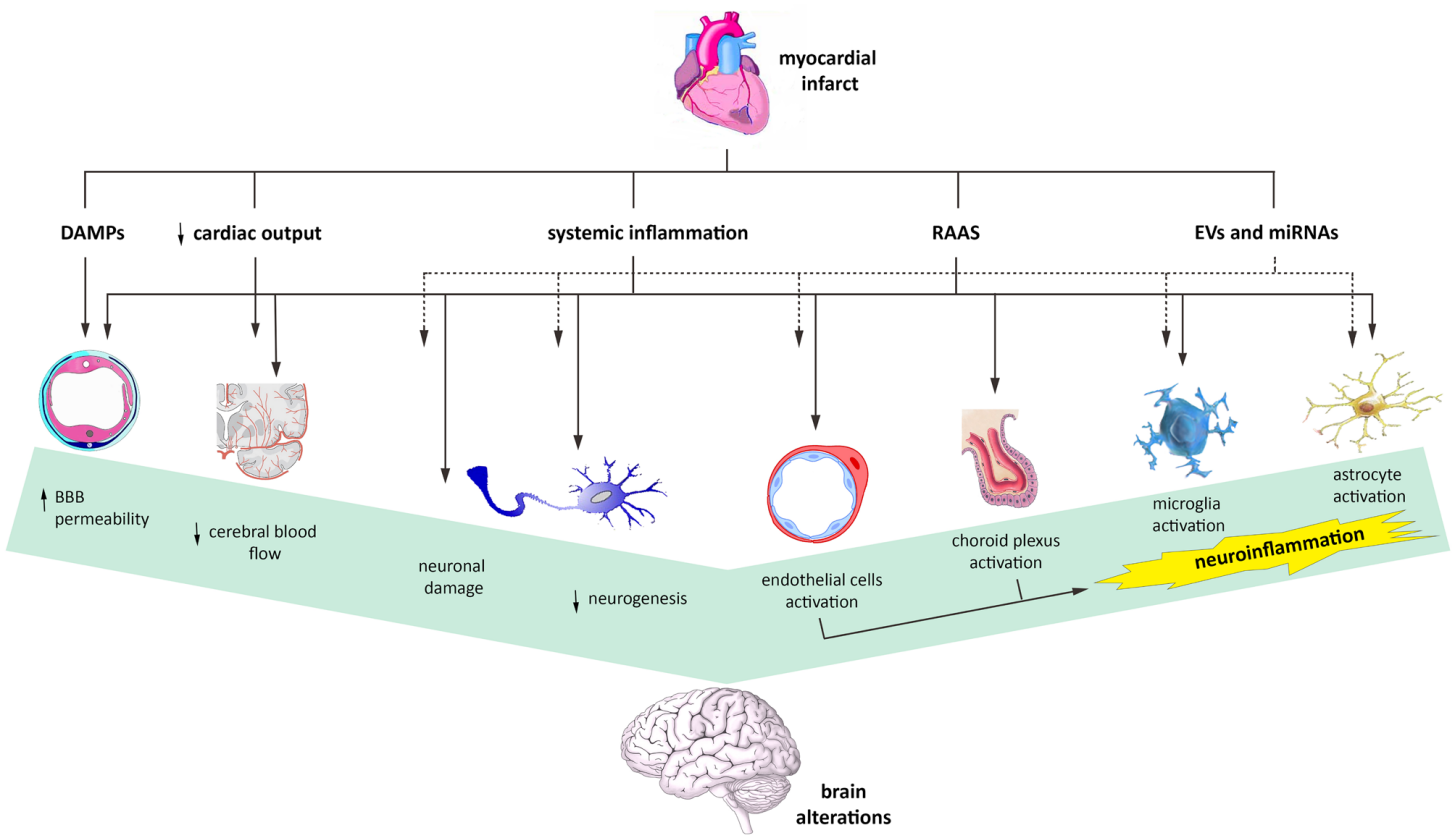
Mechanisms and cerebral effects of heart–brain interaction after MI. Brain alterations after MI may be caused by several mechanisms, including systemic inflammation, activation of the renin–angiotensin–aldosterone system (RAAS), circulating cardiac-derived DAMPs, EVs, and miRNAs, and reduced cardiac output

Figure 1 summarizes all the mechanisms discussed.

### Immunoresponse and inflammation

It is widely documented that the immune system and inflammatory processes are activated following MI [29]. After MI, dying cardiomyocytes and the other cells populations in cardiac tissue release damage-associated molecular patterns (DAMPs) that can be recognized by pattern recognition receptors (PRRs) expressed by several immune cells, including neutrophils, monocytes/macrophages, and dendritic cells. In particular, cardiac resident CCR2^+^ macrophages are activated by DAMPs through binding with the toll-like receptor (TLR) 9, and, as a result, they increase the expression of the chemokine (C-X-C motif) ligand CXCL2 and CXCL5, stimulating the migration of neutrophils into cardiac ischemic tissue [30]. In turn, releasing their granule contents, neutrophils increase vessel permeability and promote, together with activated macrophages, the migration of monocytes to the site of inflammation [31]. The subsequent excessive production of the reactive oxygen species (ROS) and impairment of anti-oxidant system as well as enhanced production of matrix metalloproteinases (MMPs), pro-inflammatory cytokines, and chemokines, further aggravate inflammation [32]. Experimental and clinical studies showed that also the adaptive immune cells contribute to inflammation following MI. Indeed, lymphocytes, especially CD4^+^ T cells, are activated after acute coronary syndrome (ACS) and MI [33–35], and B cells can influence the monocyte migration after MI producing the chemokine CCL7 [36].

The activated immunoresponse and the inflammatory processes, necessary for cardiac remodeling and scar formation, are localized not only in the cardiac tissue, but also at the systemic level, as demonstrated by the high levels of circulating cytokines, such as TNF-α, IL-1, and IL-16, which persist for several weeks after the ischemic event and correlate with deteriorating cardiac function and increased mortality [37–41]. The circulating cytokines may evoke a cascade of events in the cerebral circulation, including thrombus formation [16]. In parallel, it has been suggested that systemic inflammation induce neuroinflammation, namely, increasing pro-inflammatory cytokines in the brain, within few minutes from MI [40, 42], and this inflammatory condition may persist even longer, approximately 6–8 weeks [40], after the initial peripheral inflammation has subsided [43]. The increased levels of cytokines in the brain may be due to the passage of peripheral cytokines through the blood–brain barrier (BBB). However, substantial increase in hypothalamic cytokines early after MI is not easily explained since cytokines are too large to readily cross the BBB. Thus, active transport of peripheral pro-inflammatory cytokines across the BBB or cytokines-mediated endothelial leakage and altered BBB permeability could be the possible routes [43, 44]. In this context, it was recently demonstrated that the pro-inflammatory DAMP, high mobility group box 1 (HMGB1), whose serum concentration increases early after the acute MI [45], dramatically enhances permeability in primary human brain microvascular endothelial cells and in human cerebromicrovascular endothelial cell line, a widely used model of human BBB in vitro [46]. This BBB alteration is matched by a significant downregulation of the zona occludin-1 (ZO-1) expression at intercellular at tight junctions [46]. Cytokines transport across the BBB could also be mediated by circulating EVs which are enriched with pro-inflammatory cytokines after MI [47]. Otherwise, the high level of pro-inflammatory cytokines may be originated from an increase in local production, but the underlying mechanism is not clearly elucidated. One hypothesis has suggested that pro-inflammatory cytokines upon crossing BBB could induce PGE2 production in endothelial cells of the cerebral blood vessels [48], leading to an increase in cerebral cytokine production. Furthermore, circulating cytokines could themselves stimulate microglia and astrocytes to produce cytokines. Another hypothesis suggested that elevated levels of angiotensin II and aldosterone following MI could initiate inflammatory response through induction of ROS [48]. The available evidence suggests that neuroinflammation after MI is a complex process in which several actors are involved in its occurrence and that integrates cellular and molecular responses, involving different cellular lineages (see “Neuroinflammation”).

### EVs

EVs are nanometer-sized, lipid membrane-enclosed vesicles released by cells into the extracellular space to facilitate intercellular communication in diverse cellular processes [49]. EVs provide a unique mode of long-range delivery of lipids, metabolites, and proteins as well as ribonucleic acids (RNA) and deoxyribonucleic acids (DNA) from donor cells to distant recipient cells. A key role in regulating the EVs-mediated interactions is played by the membrane-bound signaling proteins of EVs, which interact with the extracellular environment determining the cell types they target. EVs have been traditionally subdivided into three major classes according to their diameter: exosomes (20–150 nm), microvesicles (also named microparticles; 100–1000 nm), and apoptotic bodies (> 500 nm) [50], but they could be often classified according to their surface proteins.

A growing number of experimental and clinical studies found that the level of microvesicles (MVs) in patients with coronary heart disease (CHD) increased significantly [47, 51, 52]. In particular, leukocyte-derived (CD45^+^lMVs), endothelium-derived (CD31^+^CD42^−^eMVs), platelet-derived (CD31^+^CD42^+^pMVs), erythrocyte-derived (CD235a^+^ ErMVs), and annexin-V^+^ MVs significantly increased in plasma of patients with subgroups of CHD, including stable angina (SA), unstable angina (UA), and MI (NSTEMI and STEMI) [53–55]. However, it is still controversial which EVs subpopulations are most useful for diagnostic or prognostic purposes. A recent meta-analysis, including 599 participants (137 healthy subjects, 148 patients with SA, 147 patients with UA, and 167 patients with MI), found that the level of MVs, especially CD31^+^CD42^−^ and CD144^+^ eEVs, was higher in patients with CHD than in healthy subjects and had an increasing trend with the degree of CHD: SA < UA < MI [56]. Moreover, an increase in cardiomyocyte-derived EVs was found in plasma samples of STEMI patients, as well as in mice subjected to permanent left anterior descending (LAD) artery ligation [52]. EVs released from cardiomyocytes under pathophysiological conditions may convey “danger or inflammatory signals” to other cells. In vitro, hypoxia-induced released of EVs carrying TNF-α [57] and heat shock protein 60 (HSP60), a ligand of TLR4, which activates the innate immune response [58]. In mice with permanent LAD artery ligation, cardiac EVs were transiently accumulated in the infarcted heart, with a peak between 15 and 24 h post-MI, and originated mainly from cardiomyocyte (caveolin-3^+^; Troponin T^+^), cardiac fibroblast (CD90.2^+^), and endothelial cells (CD31^+^CD41^−^), while only a small population of leukocyte-derived CD45^+^ EVs was detected. These EVs were taken up by infiltrating monocytes/macrophages and regulated local inflammatory responses, leading an increased release of IL-6 and chemokines CCL2 and CCL7 [59]. After MI, circulating EVs could be also enriched with pro-inflammatory cytokines. Indeed, 24 h after MI in rats, plasma-derived EV were enriched with pro-inflammatory cytokines IL-1α, IL-1β, and Rantes. When added to the perfusates of isolated-perfused hearts, these EVs induced cardiomyocyte death and cardiac dysfunction through activation of the TLR4/NF-κB axis, whereas circulating EVs from healthy rats did not [47].

To date, it is widely accepted that EVs released by multiple cell types in response to MI participate both in the inflammatory injury and in tissue repair, but it is likely that they may influence other organs, such as the brain.

EVs were suggested as important signals mediating heart–brain interactions [1]. In fact, EVs released from the heart in normal or pathological conditions could influence both heart and brain, since some of molecular mechanisms and signaling pathways involving EVs were similar in MI and stroke. Although they are two distinct pathological conditions affecting different organs and type of cells, circulating EVs in patients after MI and stroke showed several similarities in proteins and microRNAs (miRNA) [60]. In details, these EVs have in common 14 proteins which were absent in healthy controls, such as apolipoprotein L1 (APOL1) and apolipoprotein C (APOC); involved in lipid metabolism; and complement C4 (C4) and C-reactive protein (CRP), factors, and activators of the complement system, which is activated after stroke and MI and contributes to tissue injury after ischemia. However, the hypothesis of circulating EVs as possible mediators of heart–brain axis was supported by two studies demonstrating that their passage through the BBB is very low in non-pathological conditions, but it was increased by orders of magnitude after chronic or acute systemic inflammation [61, 62]. Upon reaching the brain, likely via adsorptive-mediated transcytosis, EVs were able to transfer functional genetic material leading to concomitant changes in miRNA content of the receiving cells. Of note, erythrocyte-derived EVs in presence of systemic inflammation or obtained from Parkinson’s disease patients provoked an increase in microglial inflammatory responses [62]. In line, a more recent study demonstrated that peripheral circulating inflammatory exosomes induce neuroinflammation also in absence of systemic inflammation. In details, serum-derived exosomes purified from LPS-challenged mice or from mice fed high-fat diet induce microglial and astrocytic activation and increase the expression of inflammatory cytokines in the brain of recipient mice. The authors suggested that ependymal cells in the third and lateral ventricles may be the main entry sites via the blood cerebral spinal fluid brain barrier (BCSFB) for the exosomes to translocate into brain parenchyma, where are primarily taken up by microglial cells [63].

**Fig. 2:**
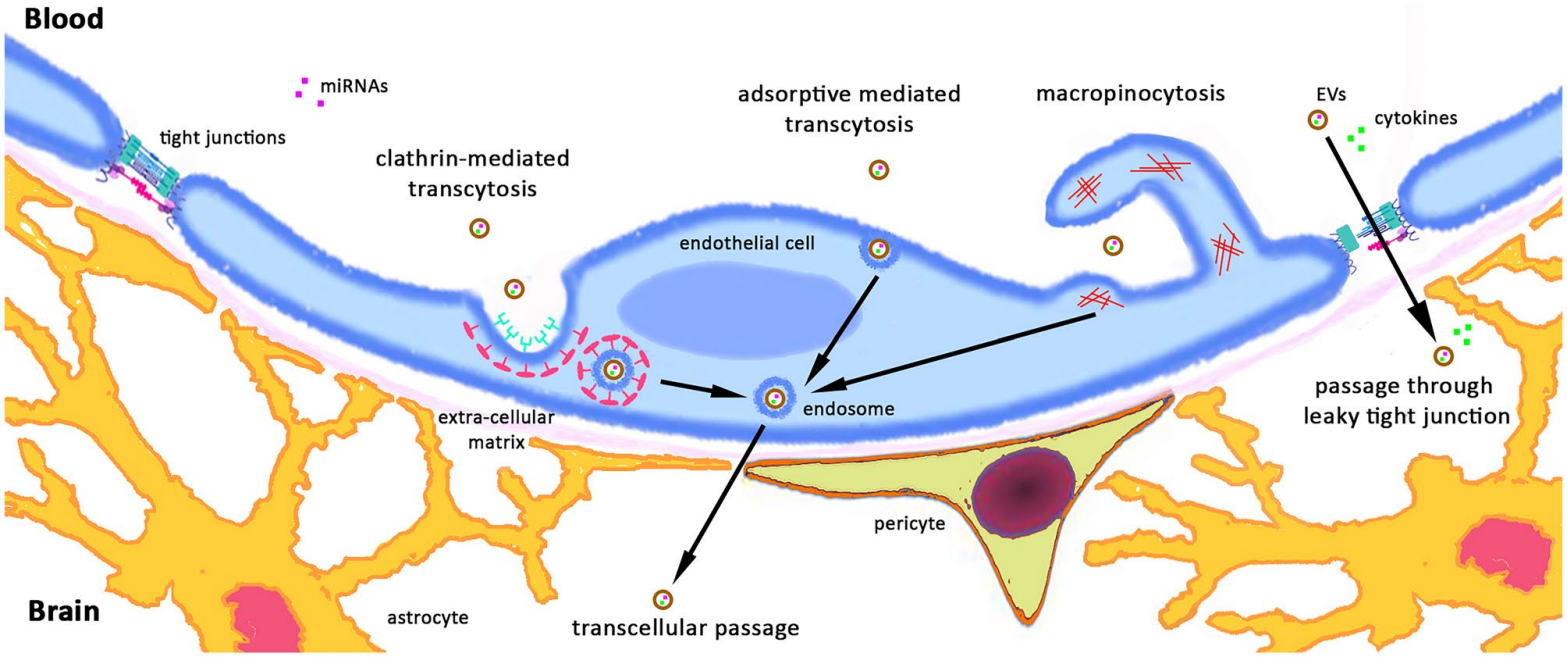
Potential pathways of cytokines, miRNAs, and EV passage across the BBB. Cytokines and miRNAs could cross BBB through EV-mediated transcellular routes, such as macropinocytosis, adsorptive mediated transcytosis, and clathrin-mediated transcytosis. In addition to transcellular routes, the breakdown of tight junction by DAMPs or inflammatory mediator may increase the permeability of cytokines and EVs at the paracellular route

The mechanisms by which EVs cross the BBB are not fully elucidated. An endocytic, transcellular mechanism was reported to mediate the enhanced exosome transport through the BBB elicited by TNF-α in an in vitro BBB model [64]. Other studies suggested that clathrin-mediated transcytosis may play a role in EVs crossing the BBB [65, 66]. Finally, adsorptive mediated transcytosis and macropinocytosis were proposed as additional transcytotic mechanisms at the BBB that allow internalization of EVs without requiring them to be coated with specific transportation molecules [67, 68] (Fig. 2). Peripheral inflammation may also influence brain homeostasis through the activation of choroid plexus epithelium (CPE) and the subsequent release of choroid plexus-derived extracellular vesicles. In mice treated with LPS, EVs were secreted by CPE into the cerebrospinal fluid (CSF) and were taken up by astrocytes and microglia, which in turn respond with an inflammatory program. As a confirmation of the role of CPE, the effects of the peripheral inflammation-induced EV production by the CPE cells were reversed by the injection of an exosome inhibitor, and this was reflected by reduced upregulation of inflammatory genes [69]. However, we cannot rule out that systemic inflammation may stimulate the release of EVs by peripheral immune cells, which could directly cross the BBB through leaky tight junction, and target brain cells.

Taken together, all these data suggest that circulating EVs released by peripheral organs under pathological conditions, including MI, could influence brain responses. However, to our knowledge, there are no studies that have assessed the cerebral effects of circulating EVs released after MI. Beyond reasonable hypotheses, future studies are needed to demonstrate whether circulating EVs is one of mechanisms through MI influences cerebral behavior.

### miRNAs

MicroRNAs are bioactive small non-coding RNAs, which interact with the complementary sequences in the 3′ untranslated region (3′UTR) of protein-coding mRNAs, resulting in the inhibition of protein translation or mRNA degradation [70]. miRNAs are secreted into the extracellular space through three main mechanisms: (1) direct excretion from the cell upon binding to RNA-binding proteins, (2) budding off the cells through MVs formation, or (3) packaged into multivesicular bodies and released from the cells as exosomes [71].

miRNAs are involved in a myriad of biological processes, including proliferation, apoptosis, metabolism, differentiation, epithelial-to-mesenchymal transition, regulation of insulin secretion, division of stem cells, embryonic development and pattering, fetal growth, and immune system, including resistance to viral infection [72]. miRNAs may have cell-type-specific or tissue-specific expression patterns or may be expressed ubiquitously, but their expression could change in spatial as well as in temporal manner in pathological conditions, suggesting miRNA as potential biomarkers [73, 74].

Significant changes of miRNA expression in peripheral total blood samples of patients with MI were reported. Although 121 different miRNAs were observed to be dysregulated [75], robust evidence was found only for miR-1, miR-133a/b, miR-208a, and miR-499, whose serum levels increased in humans and animals following MI [76–79]. All these four miRNAs are regarded as heart-specific miRNAs [73] and found in plasma carried by exosomes (miR-1, miR-208, and miR-499) or as free-circulating compound (miR-133) [79].

**Fig. 3:**
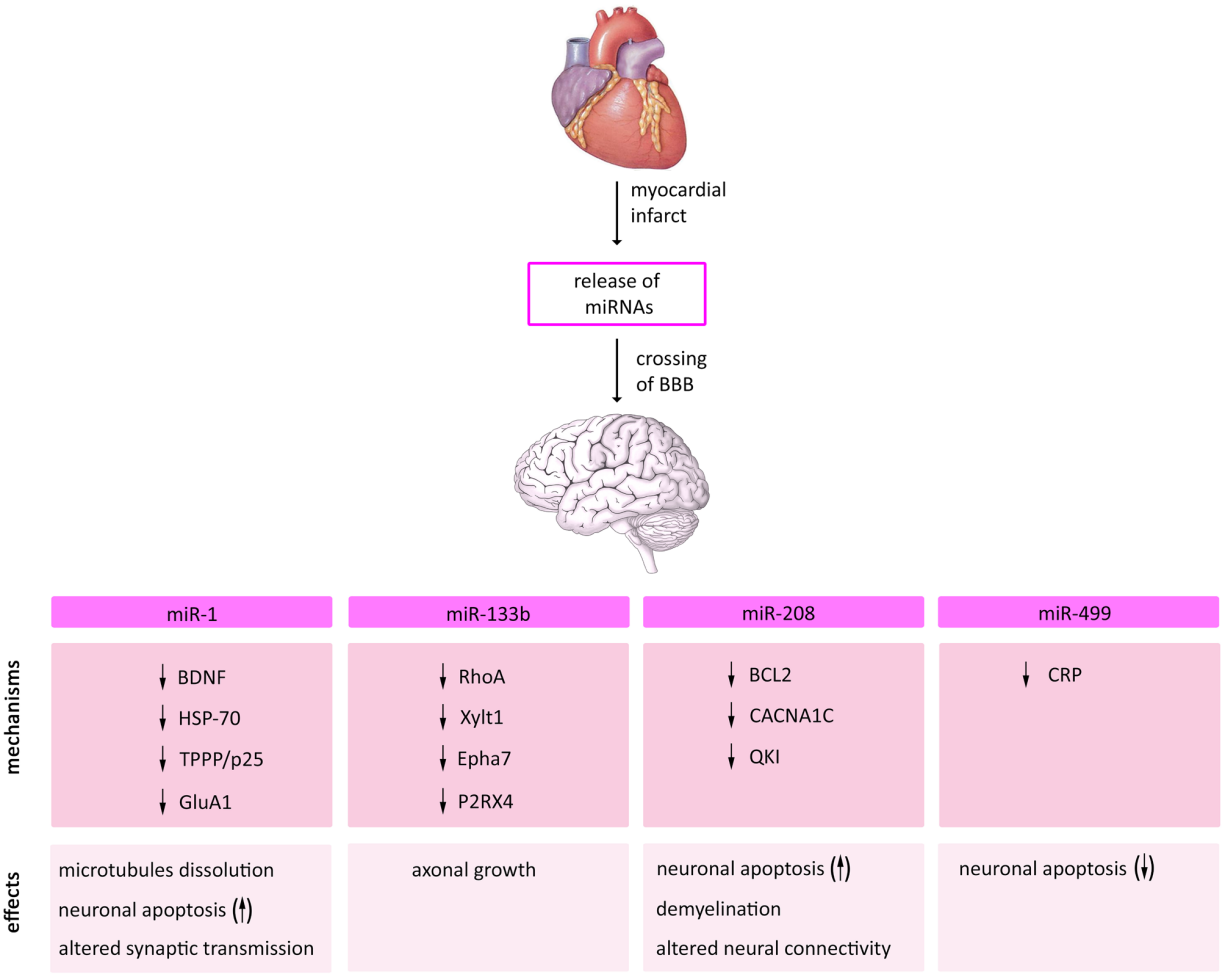
The possible role of miRNAs released from the heart after MI in mediating cerebral effects. An overview of the molecular mechanisms and cerebral effects of the miRNAs whose expression are increased in the peripheral blood of patients with MI and for which it has been reported an involvement in cerebral physiological and pathological conditions

In addition to modulating the signaling pathways after MI, several studies showed that these miRNAs are involved in cerebral physiological and pathological conditions, suggesting a possible mechanism through which MI affects the brain (Fig. 3). Circulating miRNAs are localized in MVs or bind to other plasma components such as high-density lipoprotein (HDL) particles and RNA-binding proteins [80]. Several evidences suggested that circulating EV-associated miRNAs are able to cross the BBB using exosomes [81–83]. However, more research must be done to elucidate how circulating cardiac-derived miRNA, in particular EV-free circulating miRNAs, cross BBB after MI.

miR-1 is specifically expressed in adult cardiac and skeletal muscle tissues, and the increase of its serum level after MI suggests a necrotic death of cardiac myocytes as source [84]. Recently, it was proven that miR-1 might control the generation of synapses, brain growth, learning, and memory through regulation of brain-derived neurotropic factor (BDNF) and impact on target genes [85]. miRNA-1 also played a role in the damage induced by hypoxia in neurons affecting the expression of HSP-70 and consequently mediating hypoxia-induced apoptotic insults via an intrinsic Bax–mitochondria–caspase protease pathway [86]. In line, miRNA-1 knockdown by injections of anti-miR1 reduced the infarct volume in transient middle cerebral artery occlusion (MCAO) models induced by endothelin-1 in female rats [87] and by intraluminal filament in male rats [88], likely modulating the IGF signaling pathway. Two recent studies have clearly demonstrated the possibility that the heart may affect the brain through miR-1. Indeed, transgenic mice with cardiac-specific over-expression of miR-1 showed cognitive impairment that may be associated, at least in part, with the downregulation of BDNF expression in the hippocampus. The authors reported also an increased expression of miR-1 in the blood and hippocampus, although the expression of primary miR-1 was not changed. The latter data strongly suggested that circulating cardiac-derived miR-1 is very likely transport from the blood to brain, leading to regulation of cerebral target genes such as BDNF [81]. In mice with permanent LAD ligation, MI induced an increase of miR-1 levels in blood and hippocampus, likely originated from infarcted heart, leading neuronal microtubule damage and a decrease in the tubulin polymerization, inhibiting protein TPPP/p25 expression in the hippocampus. These changes were prevented by the selective knockdown of miR-1 in the hippocampus [82]. Relevantly, in transgenic mice with cardiac-specific over-expression of miR-1 and subjected to permanent LAD ligation, MI induced a reduction of learning, memory, and efficiency of synaptic transmission, likely through downregulation of BDNF, GluA1 subunit of the AMPA receptor, and dephosphorylation of GluA1. All these effects were prevented by the injection of a miR-1 antisense inhibitor in the CA1 area of the hippocampus [89]. Taken together, these results strongly indicate that the cardiac-originated miR-1 is a direct actor of brain dysfunction after MI.

Instead, miR‐133b has been suggested to regulate neurite outgrowth. Indeed, inhibition of miR‐133b expression by synthetic antisense oligonucleotides resulted in impaired locomotor recovery and reduced regeneration of axons after spinal cord injury (SCI) in adult zebrafish. The authors showed that miR-133b targets the Ras homolog gene family member A (RhoA), an inhibitor of axonal growth, as well as other neurite outgrowth‐related molecules [90]. Furthermore, in mice with SCI, the enhanced expression of miR-133b by lentiviral vector injection improved locomotor recovery by downregulation of the expression level of RhoA and chondroitin sulfate proteoglycans, and by decrease of infiltrating microglia/macrophage [91]. The same authors showed that transfection of miR-133b stimulated neurite outgrowth in cultured hippocampal neurons, likely decreasing the expression of RhoA, but also of xylosyltransferase 1 (Xylt1), an enzyme involved in the synthesis of chondroitin and dermatan sulfates, ephrin receptor A7 (Epha7), a key regulator of axon guidance, and purinergic receptor P2X ligand-gated ion channel 4 (P2RX4). In cell culture models of Parkinson’s disease, the overexpression of miR-133b ameliorated the MPP^+^-induced axon degeneration, blocking the MPP^+^-induced decrease in the Bcl-2/Bax ratio and increasing the activity of the pro-survival kinase Akt (p-Akt) [92], and was involved in the downregulating of α-synuclein [93]. Finally, the neuroprotective effect of miR-133b was also observed in a rat model of cardiac arrest [94]. In details, miR-133b incorporated in EVs, which were released from transplanted bone marrow mesenchymal stem cells (BMSCs), promoted survival of neuronal cells via regulation of JAK1 and AKT-GSK-3β-WNT pathway.

A recent study suggested that increased expression of miR-208 may augment susceptibility to schizophrenia by simultaneously conferring susceptibility to apoptosis and altering neural processing and connectivity through the suppression of BCL2 and calcium voltage-gated channel subunit alpha1 C (CACNA1C), respectively [95]. Furthermore, miR-208 reduced the expression of the RNA-binding protein quaking (QKI), whose suppression commonly contributes to demyelination of the neurons [96].

The serum level of miR-499 was markedly increased in the traumatic brain injury (TBI) patients compared with the healthy subjects and was associated with injury severity and clinical outcome, suggesting that miR-499 may serve as biomarker for the diagnosis and progression monitoring of TBI and that they may be involved in TBI pathogenesis [97]. However, the cerebral expression levels of miR-499-5p were gradually decreased after perinatal hypoxic-ischemic encephalopathy (HIE) in rats, while miR-499-5p injection significantly improved long-term neurological function recovery and decreased HIE-induced brain injury, reducing apoptotic neurons in the hippocampus, the infarct size, and level of CRP [98].

## Cerebral alterations as a consequence of MI

### Neuroinflammation

**Fig. 4:**
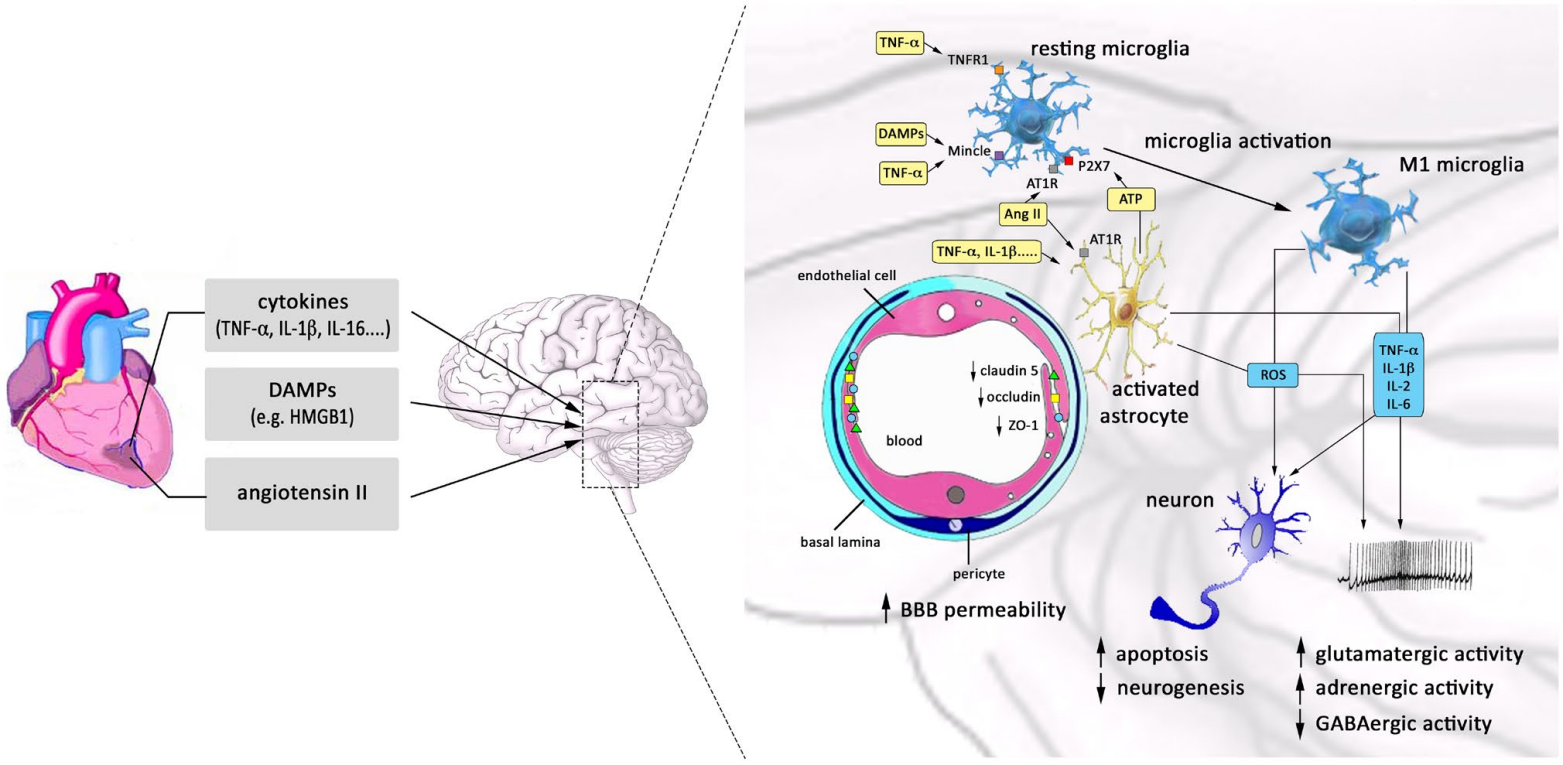
Summary of mechanisms for neuroinflammation after MI. Circulating pro-inflammatory cytokines, DAMPs, and angiotensin II (Ang II) released from the ischemic heart reach the brain through the blood. Here, they can compromise integrity and enhance permeability of BBB, reducing the expression of junctional proteins such as ZO-1, occludin, and claudin-5. Ang-II and pro-inflammatory cytokines induce the activation of astrocytes and stimulate resting microglia to assume the pro-inflammatory M1 phenotype, which is induced also by the excessive release of ATP from activated astrocytes. In turn, M1 microglia and activated astrocytes produce large amounts of cytokines and ROS, which perpetuate neuroinflammation and lead to enhanced neuronal apoptosis and decreased neurogenesis. Furthermore, they produce an imbalance between excitatory and inhibitory neurotransmission, potentiating excitatory (glutamatergic and adrenergic) currents and inhibiting GABAergic currents

Neuroinflammation, defined as an inflammatory response within the brain, is mediated by the production of cytokines, chemokines, and ROS, which are produced by microglia, astrocytes, peripherally derived immune cells, and endothelial cells (Fig. 4). It is widely recognized that MI is able to induce neuroinflammation, which may persist even long after the initial peripheral inflammation has subsided [43], approximately 6–8 weeks after MI in the hypothalamus of rats [40].

A recent study showed that MI, obtained in mice by permanent LAD coronary artery ligation, induced a cerebral increased expression of TNF precursor protein, a more than doubled TNFR1 expression, and almost 70% decline in TNFR2 expression, suggesting a shift toward a more pro-inflammatory state [41]. Increased brain cytokines can affect several processes involved in neuronal function, including apoptosis, oxidative stress, and metabolic processes [99]. In addition, neuroinflammation is thought to be a key for Alzheimer’s dementia progression [100], and it is able to influence behavioral aspects, including anxiety, cognitive deficit, and depression [101]. In this regard, sex and estrogen level could influence neuroinflammation and depression-like behavior. A recent study in male and female rats with permanent LAD artery ligation showed that cytokines (TNF-α, IL-1β, IL-2, and IL-6) increased significantly in the prefrontal cortex of MI male rats, while no changes were found in MI female rats [102].

Although circulating cytokines could cross BBB, as reported in “Immunoresponse and inflammation,” recent data indicate that the increase in pro-inflammatory cytokines involves the activation of cerebral cells [40, 103]. In response to injury or inflammation, context-specific signals can shape both astrocyte and microglial responses. In this context, for example, it has been shown that the blocking of TNF-α by its antagonist etanercept or by its genetic deletion causes a reduction in microglia activation, pointing the pivotal role of this cytokine in glial activation in mice with permanent LAD coronary ligation [104]. In light of these evidences, several studies have analyzed the effect of MI on microglia and astrocytes.

Infarcted rat, subjected to permanent LAD coronary ligation, showed microglial activation in the paraventricular nucleus (PVN) of the hypothalamus, in periaqueductal grey (PAG), in rostral ventrolateral medulla (RVLM), in nucleus tractus solitarius (NTS), and in area postrema (AP) [105, 106] during the late post-ischemia phases, but not in the earlier phase (within 1 week) [107]. In contrast, an early activation of microglia was observed in more recent studies [108–110]. In particular, cardiac ischemia/reperfusion (I/R) induced a shift of microglia phenotype from the beneficial M2 to the inflammatory harmful M1 at the end of 120 min of reperfusion period. Indeed, cardiac I/R induces an increase in Iba-1 positive cells, CD11b^+^/CD45^+high^ microglia, and microglial dendritic volume, while filament length and dendrite complexity significantly decreased, suggesting that microglia tends toward an ameboid shape or M1 phenotype, indicating both activation and acquisition of phagocytic properties. These effects are, almost in part, mediated by the proprotein convertase subtilisin/kexin type 9 (PCSK9). Indeed, its cerebral expression is increased during cardiac I/R injury, and its inhibition reduces neuronal inflammation possibly because of a reduction of systemic inflammation [109].

However, the microglial activation was relatively selective within the PVN, since changes in the phenotype of microglia and a re-arrangement of its cytoskeleton occur in the PVN but not in the adjacent areas and in the cortex of MI rats [107, 111, 112]. Taken together, these results seem to suggest that, unlike other peripheral inflammatory conditions such as inflammatory bowel disease, MI not induces a generalized increase in the permeability of the BBB that could explain the activation of microglia, but only in selected brain regions that are centers for cardiovascular autonomic regulation [43, 103, 105]. This hypothesis has been challenged by recent studies showing a decreased expression of BBB tight junction proteins, claudin-5, and occludin, in whole brain [110], and an increased number of hypertrophic and dystrophic-like microglia in the prefrontal cortex, thalamus, and hippocampus of rats with permanent LAD coronary ligation [113].

Analyzing the results, another consideration to take into account is the responsiveness of microglia to MI that seems different between rats and mice. Contrary to mice, in rats, microglia remain active for a long time after MI, up to 16 weeks [107], whereas, after 18 days from permanent LAD artery ligation, in MI mice was reported alternative activated hyper-ramified microglia in PVN, that may represent a state of increased alertness rather than reflecting neuroinflammation [114]. Similarly, in a study using permanent ligation of the coronary artery in mice, no changes in reactive microgliosis were observed 3 months post-MI [115]. This discrepancy could be explained by the phasic pattern of microglia activation or the models used in mice. Indeed, a recent study, conducted in a mouse model of permanent cardiac ischemia, observed biphasic activation of microglia, with activity peaks after 1 and 8 weeks of ischemia, interspersed with a decline to 4 weeks [2]. The authors state that the systemic inflammatory response to acute MI may serve as the primer for the subsequent reoccurrence of neuroinflammation in the chronic phase of MI leading to heart failure.

It was speculated that the release of cytokines from activated microglia could stimulate neurons of PVN, contributing to the elevated sympathetic nerve activity seen in chronic heart failure [116]. In this context, two recent studies in rats with permanent LAD artery ligation showed that MI activates microglia which subsequently release cytokines in the PVN. In the first study, MI induced production of ATP which activates microglia through P2X7 receptor acutely promoting the synthesis of TNF-α and IL‐1β in the PVN [117]. The second study showed that MI induces, within the PVN, microglia stimulation through activation of macrophage-inducible C-type lectin (Mincle), a receptor primarily expressed in microglia which recognizes the DAMPs derived from dead cells [118], further causing sympathetic hyperactivity via NLRP3/IL‐1β dependent pathway [108]. The authors speculated that SAP130, a spliceosome-associated protein 130, and cytokines such as TNF‐α and IL‐6 derived from MI crossing through the BBB, could activate Mincle in microglia, resulting in pro‐inflammatory cytokines release and broader inflammatory response. However, how cytokines could affect sympathetic activity post‐MI is unknown. Previous studies have demonstrated that TNF-α and IL‐1β secretion promotes expression of activated nuclear transcription factor kappa B (NF‐kB) and ROS, which could enhance glutamatergic and adrenergic excitatory transmission and attenuate GABAergic inhibitory activity in the PVN [119, 120].

It is well documented that microglia activation occurs in animal models of stress-induced depression [121]. Thus, the activation of microglia was suggested to be involved in depression-like behavior developing in the chronic phase after MI. In the hippocampus of mice undergoing cardiac I/R, an increase in the number of microglial cells has been observed, and it is associated with a worsening of performance in learning tests involving hippocampus functionality [122]. Similarly, after permanent LAD coronary ligation, an increase in microglia and its activation, associated with a worsening of cognitive function, has been observed in the dentate gyrus of the hippocampus in mice [104, 114], and in dentate gyrus of the hippocampus and in the PVN of the hypothalamus in rats [106, 123]. It is likely that microglia activation, increasing local secretion of pro-inflammatory cytokines, could exacerbate neuronal activity [103, 105] and induce neuronal death [112] leading to behavioral signs of depression.

In additions to microglia, MI could activate also astrocytes. As observed in the amygdala of rats, MI induced morphological changes of astrocytes starting from 3 days after permanent LAD artery ligation, and 14 days post-MI, several GFAP^+^ astrocytes showed hypertrophied cytoplasm and highly ramified processes [123]. This evidence was confirmed by a more recent study showing this typical “activated” morphology of GFAP^+^ cells in the PVN of rats at 1 day after permanent LAD. Relevantly, astrocytes inhibition, by PVN injection of fluorocitrate, reduced the MI-induced expression of pro-inflammatory cytokines (TNF-α and IL-6), neuronal activation, and ventricular arrhythmia occurrence, and improved ventricular electrical instability [124]. Similarly, an early astrocytic activation was observed in rat model of cardiac I/R, as suggested by the increased numbers of GFAP^+^ cells, dendritic volume and complexity, and decreased filament length at the end of the 120 min of reperfusion period [109]. As for microglia, a recent study suggested that astrocytes regulate sympathetic activity via the release of ATP in the RVLM of rats with MI [125]. On the contrary, in MI mice with permanent LAD artery ligation were not observed an increased density of astrocytes in the hippocampus [114] and in the rostral ventrolateral medulla [126] after MI. This discrepancy in the effect of cardiac ischemia on astrocytes activation appears to be species-related, as seems also for microglia. Overall, these results suggest that astrocyte activation is involved in neuroinflammation and in the early phase of cardiac sympathetic hyper-activation following MI. This action seems to be mediated by the angiotensin II type 1 receptor (AT1R), which is weakly expressed in astrocytes under basal condition. The GFAP-specific AT1R deletion in mice with permanent LAD artery ligation inhibited the MI-induced upregulation of brain AT1R, despite the preservation of neuronal AT1R expression, and enhanced central sympathetic outflow, probably by inhibiting reactive oxygen species (ROS) [126].

### Neuronal apoptosis, neuronal plasticity, and impaired neurogenesis

Inflammation is often associated with enhanced pro-apoptotic processes and altered neurogenesis, and these phenomena happen also in the brain after MI and could contribute to the development of the depressive-like behavior.

In rats subjected to cardiac I/R, MI acutely decreased P13K activity and increased Bax/Bcl-2 ratio, caspase-3 activity, and numbers of TUNEL-positive cells in the amygdala, suggesting a possible link with the major depressive disorder observed following MI [127]. The same result on a caspase-3 activation in the amygdala of rats with cardiac I/R was observed in a recent study [128]. Interestingly, in the same animal model, an increased Bax/Bcl-2 ratio was observed in the hypothalamus and prefrontal cortex, but not in the amygdala and hippocampus, at subacute phase after MI. Together with the absence of enhanced caspase-3 activity, these results suggest a caspase-3 independent mechanism or different time of apoptosis activation in these cerebral structures after MI [129], as demonstrated by another study [130].

It was suggested that the MI-induced brain apoptosis may be ascribed to oxidative stress, mitochondrial dysfunction, and enhanced permeabilization of the mitochondrial outer membrane. The latter may lead to the release of pro-apoptotic proteins including Bax and cytochrome c, which activates caspase cascade [110]. Another study in a rat model of cardiac I/R also showed that MI decreased the expression of the receptor-interacting serine/threonine-protein kinase 1 (RIPK1), a protein implicated in the plasma membrane permeabilization and necrotic cell death, likely due to caspase-8-mediated cleavage, shifting the cell towards apoptosis [131]. An activation of apoptosis was also observed in rats with permanent LAD artery ligation. MI induced an increased mRNA expression of caspase-3, caspase-8, and caspase-9 and Bcl-2 in the hippocampus, which were associated with anxiety-like behavior [132]. A recent study demonstrated that enhanced plasma level of TNF-α contributes to apoptosis via activation of the extrinsic pathway in the limbic system after MI. The inhibition of TNF-α by PEG sTNFRI, a soluble p55 type 1 TNF receptor, reversed the MI-induced increase of caspase-3 and caspase-8 activity in medial amygdala, dentate gyrus, and hippocampus (CA1) [133].

The role of TNF-α signaling in the MI-mediated neurodegenerative processes is also supported by evidence showing that pharmacological blocking or genetic deletion of TNF-α ameliorated the reduction of cortical dendritic spines in mice with permanent LAD coronary ligation [104]. The dendritic spine density was also found to be reduced in the hippocampus of rats with transient LAD artery ligation [109, 110]. A decreased loss of dendritic spine density was observed after inhibition of PCSK9, suggesting its involvement in neuronal damage following cardiac I/R insults [109]. On the contrary, two studies showed no neurodegeneration and neuronal death in the dentate gyrus and hippocampus (CA1) of rats [134] and mice [135] with permanent LAD artery ligation. These contrasting results may be caused by the short time scale of apoptosis, the time of sampling, or the sensibility of techniques utilized.

In addition to apoptosis, MI could also affect neurogenesis. In mice with transient LAD artery ligation, neurogenesis in the granular zone of dentate gyrus was significantly decreased both acutely and chronically after MI, potentially contributing to the cognitive decline [122]. In contrast, in rats with permanent LAD artery ligation, MI enhanced cell proliferation and neuroblast differentiation in the subgranular zone of the dentate gyrus [134], while in mice with permanent LAD artery ligation, MI did not influence neurogenesis [41, 135]. In a more recent study using mice with permanent LAD artery ligation, neurogenesis slightly decreased in the hippocampus and in the piriform cortex [114]. These discrepancies may be ascribed to difference of the model, the species, and the age of animals.

## Future prospective

As detailed in this review, the innate immune response and systemic inflammation could play a pivotal role in the development of neuroinflammation after MI. Thus, it would be rational to suppose that appropriate immunosuppressant and anti-inflammatory therapeutic strategies may have potential beneficial effects on the brain in post-MI conditions. Although there are no studies in this regard, some data obtained in different experimental or clinical contexts of systemic inflammation appear promising. In a model of chronic inflammatory disorder, as rheumatoid arthritis, the antagonism of TNF-α with infliximab reduced the infarct volume and the amount of microglia and activated macrophages in the ischemic hemisphere and improved the integrity of BBB and the neurological deficit in mice with ischemia/reperfusion (I/R) brain injury [136]. In patients with psoriasis, the antagonism of TNF-α with etanercept also reduced the circulating levels of inflammatory and cardiovascular proteins, such as TNF-α, IL-1β, IL-6, and IL-8 [137]. Moreover, the antagonism of IL-1β with canakinumab significantly reduced the evidence of residual inflammatory risk in patients with prior MI [138]. Relevantly, the systemic administration of interleukin-1 receptor antagonist (IL-1Ra) has shown to be neuroprotective and increased post-stroke neurogenesis in a murine model of atherosclerosis, obesity, and insulin resistance after cerebral ischemia, suggesting that this strategy as potential neuroprotective in patients with a raised inflammatory burden [139].

However, this anti-inflammatory strategy is far to be demonstrated safe in post-MI conditions, and instead could be contraindicated. Indeed, etanercept reduced systemic inflammation but increased platelet activation in MI patients [140], possibly leading a higher risk of cardiovascular events. In line, high doses of infliximab, a chimeric monoclonal antibody to TNF-α, increased the combined risk of death from any cause or hospitalization for heart failure (hazard ratio = 2.84) in patients with moderate-to-severe heart failure [141]. In rats, infliximab had a slight protective effect in the early hours after MI, but in the following days, it exacerbated the cardiac dysfunction, likely blocking the functions of compensatory mechanisms after MI such as cardiac remodeling, preventing tissue repair, and promoting further myocardial injury [142]. Altogether, these data suggest that a tolerable inflammatory process following MI could boost a healing procedure of heart tissue injuries and remodeling. Thus, anti-inflammatory strategies to reduce systemic inflammation after MI should be carefully balanced as they might interfere with cardiac tissue repair and healing.

In addition to cytokine antagonists, EVs could offer therapeutic chances for neuroprotection after MI, because of the low immunogenicity and toxicity, high blood circulation stability, and the unique ability of EVs to pass through the BBB. In particular, mesenchymal stem cell (MSC)­derived EVs emerge as a potential candidate. This type of EVs could play a beneficial role in both the heart and brain in post-MI conditions. Indeed, the treatment with MSC-derived EVs was shown to reduce infarct size and enhance cardiac function and geometry, by decreasing oxidative stress and activating pro-survival signaling, in several animal models of MI [143–145]. Parallel to the effectiveness at cardiac level, MSC-derived EVs exerted also neuroprotective effects. In vitro studies have shown that MSC-derived EVs increase neuronal survival and stimulate neural cell regeneration, growth, and proliferation [146, 147]. Relevantly, MSC-derived EVs shifted microglia from activated pro-inflammatory states towards homeostatic and shriveling functions after cortical injury in aged monkeys [148]. In rats with focal brain injury, human bone marrow, MSC-derived EVs attenuated neuroinflammation, decreasing the level of pro-inflammatory cytokines and chemokines, and the number of activated immune cells, such as astrocytes, microglia, and infiltrating leucocytes, including T cytotoxic cells [149]. However, the neuroprotective effects of EVs are not exclusive of MSC-derived EVs. Indeed, treatment with platelet-derived EVs increased proliferation of neural progenitor cells, induced angiogenesis, and improved general motor and cognitive functions in rats after permanent ischemic stroke [150].

Several evidence reveal that the mechanisms of neuroprotective action of EVs might involve the transfer of specific miRNAs to resident cells [151, 152]. Various types of miRNAs could be involved in these processes, including miR-133b [153]. In vitro studies have proven that EVs from astrocytes, which were treated with MSC-derived EVs over-expressing miR-133b, significantly increase neurite growth in primary cortical neuronal cultures subjected to oxygen–glucose deprivation (OGD) as compared to EVs derived from untreated astrocytes [154]. In vivo studies confirmed that EVs modulate responses after ischemic stroke by transferring miR-133b. Indeed, MSC-derived EVs improved functional recovery and exhibited increased axonal plasticity and neurite remodeling in rats with transient cerebral ischemia. These neuroprotective effects were attenuated by the knocking-down of the miR-133b level in MSC-derived EVs, while were significantly enhanced by the miR-133b over-expression [83].

However, to translate MSC-derived EVs over-expressing miR-133b from the bench to the bedside, further studies evaluating the impact on the heart–brain axis, especially on the mutual interactions of these two organs in post-MI conditions, should be performed. Indeed, the same EV-miRNA may affect different signaling pathways in the heart or in the brain, or exert different effects depending on the stage of heart disease after MI, with opposite effects on disease outcomes. An example of this possible dual effect is provided by miR-1. In the early phases after MI, when the circulating level of miR-1 is increased, the antagonism of miR-1 with a specific antagomir exerted a significant protective effect on heart function, decreasing cardiomyocyte apoptosis and alleviating myocardial fibrosis and remodeling. The enhanced expression of miR-1 by a lentiviral vector exerted instead opposite effects [155]. In line, in an animal model of I/R injury using transgenic mice over-expressing miR-1, it was observed an increase of infarct size, apoptosis, and caspase-3 expression [156]. On the contrary, miR-1 expression was decreased in failing hearts [157]. The restoring of miR-1 expression was associated with normalized sodium–calcium exchanger (NCX)-1 expression and improved cardiac function in a chronic post-MI rat model of heart failure [157]. In mice with ligation of LAD, transplantation of MSCs over-expressing miR-1 was more effective for cardiac repair and for improved cardiac function, by enhancing cell survival and cardiomyocyte differentiation, compared to the MSCs without miR-1 over-expression [158]. In rat with MI, the upregulation of miR-1 expression partially contributed to the post-transcriptional repression of hyperpolarization-activated cyclic nucleotide-gated channel (HNC) protein expression, which may contribute to the effect of spironolactone to reduce the incidence of MI-associated ventricular arrhythmias [159].

Altogether, these data suggest a dual role of miR-1 in the phases of heart disease post-MI. In the early phase of MI, miR-1 may regulate cell death and oxidative stress, while in the later phase may contribute to post-MI remodeling or function as compensatory mechanisms.

However, the neuroprotective effects of the antagonism of miR-1 in post-MI conditions were provided by solid evidence (see “Mechanisms of heart–brain interaction after MI”) [81, 82, 87, 88]. To note, the heart-specific miR-1 over-expression was shown to directly mediate brain dysfunction [81]. Indeed, the transgenic mouse model of cardiac-specific over-expression of miR-1–2 showed increased miR-1 levels not only in the heart, but also in the blood and hippocampus, and cognitive impairment. It is reasonable to assume that, after crossing the BBB, the circulating heart-derived EVs release miR-1 to cerebral resident cells, which in turn inhibit the expression of BDNF, leading to the impairment of cognition [81]. Similarly, the increased miR-1 level observed in the hippocampus of MI mice [82, 89], in spite of unchanged endogenous biogenesis [82], suggests that this increase might arise directly from the infarcted heart through EV-mediated transfer. Indeed, the inhibition of EVs biogenesis prevented the MI-induced elevation of miR-1 levels in the blood and hippocampus, and the subsequent hippocampal microtubule damage [82]. In all these three studies, the knockdown of miR-1 reversed the cerebral dysfunctions, restoring the BDNF levels or the neuronal microtubules in the hippocampus.

In summary, due to the promising results obtained in experimental studies, an application of EVs in the management of brain complications in MI patients is of great interest from the clinical point of view. Although, no clinical trial of EVs transplantation has been performed to evaluate cerebral outcome in MI patients; these results will promote the development of protocols for the use of EVs in clinical trials.

## Conclusion

Recent evidence has led to consider myocardial infarction not only a mere disease of the heart, but a more complex disease mediating pathological response of many distant organs, including the brain. Myocardial infarction has a short- and long-term deleterious impact on brain homeostasis, which plays a causative role in occurrence in anxiety, depression cognitive deficits, and stroke in MI patients. In addition to the increased coagulation and thrombosis, other factors may favor brain damage after MI. In particular, enhanced systemic inflammation and changes in EVs and circulating miRNAs pattern released from heart and blood cells could also play a role in increase the risk of stroke in patients with MI. It is conceivable that these different pathways, likely interact closely with each other, could contribute to neuroinflammation and subsequent alteration of neuronal function, including apoptosis and neurogenesis, and oxidative stress. However, since the data are obtained from limited experiments studies, future research is required to precisely identify the further possible cardiac-specific mechanisms involved in facilitating the onset, or in affecting, the evolution of stroke in patient with MI. A better understanding of interactions within the heart–brain axis will improve the strategies, including novel neuroprotective approaches, to prevent or treat the brain dysfunctions of MI patients.

## Data Availability

All data and materials are available and support the published claims and comply with field standards.

## Code availability

Not applicable. No coding in the study.
